# Identifying Adolescents at Risk for Emotional Disorders with Latent Profile Analysis: A Personalized, Transdiagnostic Preventive Intervention

**DOI:** 10.1007/s10578-024-01689-z

**Published:** 2024-04-12

**Authors:** José A. Piqueras, Raquel Falcó, Pilar Rico-Bordera, Josefa Canals, Lourdes Espinosa-Fernández, Manuel Vivas-Fernández, Luis-Joaquin Garcia-Lopez

**Affiliations:** 1 Division of Personality, Assessment and Psychological Treatment, Department of Health Psychology, Miguel Hernández University (UMH), 03202 Elche, Spain; 2 Department of Psychology, Research Centre for Behavioral Assessment (CRAMC), Universitat Rovira i Virgili (URV), Tarragona, Spain; 3 Division of Clinical Psychology, Department of Psychology, University of Jaen, Jaen, Spain

**Keywords:** Trasnsdiagnostic intervention, Emotional problems, Prevention, Resilience, Risk factors

## Abstract

It can be challenging to assign patients to the appropriate intervention programs, as risk and protective factors for developing emotional disorders are multiple and shared across disorders. This study aimed to provide a theoretical and empirical approach to identify and categorise adolescents into different levels of severity. The risk of developing emotional symptoms was assessed in 1425 Spanish adolescents (*M* = 14.34, *SD* = 1.76; 59.9% women). Latent Profile Analysis (LPA) was conducted to identify subgroups based on their emotional symptom severity, risk, and resilience factors. Results revealed four profiles: at low risk (emotionally healthy), moderate risk (for selective interventions), high risk (for indicated interventions), and severe risk (for clinical referral). Older age and especially female gender were predictors of higher risk clusters, and there were differences in the levels of psychopathology and health-related quality of life across clusters. Identification of at-risk adolescents for emotional disorders by means of LPA may contribute to designing personalised and tailored prevention programs that match adolescents’ specific needs.

**Supplementary Information:**

The online version contains supplementary material available at 10.1007/s10578-024-01689-z.

Emotional disorders are the leading cause of the global health burden, with depressive and anxiety disorders being the most significant contributors, according to the World Health Organization [69]. Also among children and adolescents, anxiety and depression are the most prevalent mental health disorders [50, 58]. Moreover, the presence of subclinical emotional symptoms increases the risk of developing mental disorders in the future [14, 58], and the COVID-19 pandemic seems to have further exacerbated this scenario, particularly in adolescents [62]. Recent research points out that evidence-based preventive interventions can reduce emotional disorders and the risk of developing clinical disorders [18, 20, 28, 29, 56, 64].

In the context of public health, preventive interventions are classified as universal, selective, and indicated [extracted from [69], p. 17; adapted from Fusar-Poli et al. [16] and Gordon [24]]. Transferring this classification to mental health interventions, and specifically to the prevention of emotional disorders, the selective approach intervenes on population subgroups who have an increased risk of developing these disorders due to the presence of synergistic risk factors, whereas the indicated approach focuses on population subgroups with minimal but detectable symptoms of an emotional disorder [61].

## Transdiagnostic Risk and Protective Factors Underlying Emotional Disorders

The onset and development of emotional disorders are the result of a combination of different risk and protective factors, such as personal, family, and school risk factors, as well as genetic and environmental variables and their interaction [12, 60]. However, the identification of these protective and resilience factors and knowledge of shared risk/protective factors remains limited [16]. Furthermore, the fact that common factors (e.g., early traumatic experiences) share the risk of different emotional disorders (anxiety, depression) and other related disorders (e.g., obsessive–compulsive, stress, and trauma, or eating behaviour) [5, 15, 26, 63] provides an important transdiagnostic approach to optimise preventive efforts [57].

In this regards, at least three reviews and meta-analytic reviews have delved into the relationship between risk and protective factors. Lynch et al. [36] identified key psychological, socio-environmental, and biological risk factors. Hazzard et al. [25] indicated that there are several transdiagnostic variables that act as risk or protective factors for emotional psychopathology from the Hierarchical Taxonomy of Psychopathology model (HiTOP model [32]), which would include the psychopathological domains of depressive, anxious and eating disorders. Thus, worry about mistakes and self-esteem were the only risk and protective factors, respectively, identified as statistically significant in these disorders [33]. Finally, Hogg et al. [26] concluded that psychological trauma is a transdiagnostic risk factor across different diagnostic criteria and spectra.

In addition, the risk and resilience framework suggests that protective factors are linked to patterns of risk, and vice versa [70]. This means that risk and resilience are intertwined —focusing on one includes the other. Some authors argue that resilience could be a risk factor for one person but a protective factor for another. Others distinguish between risk-promoting and protective effects, stating that resilience is protective, but its lack may not introduce extra risk [42].

Despite this, it is well known that some of the risk factors that play an important role in the development of emotional disorders in adolescents include the following:

1. Social rejection and peer victimisation, including experiences of cyberbullying, bullying, discrimination, and exclusion. These experiences can lead to feelings of isolation, low self-esteem, and a lack of social support [5, 26, 38].
2. Exposure to trauma in childhood and stressful life events (including those related to COVID-19, such as social isolation, financial insecurity, and fear of illness) are associated with elevated risk for emotional psychopathology (Estrategia España 2050, Gobierno de España, p. 369; [38, 62]).
3. Unhealthy lifestyle habits, such as poor diet, lack of exercise, sleep problems, substance abuse, or Internet abuse. These habits can disrupt the body's biological processes, which may increase susceptibility to disorders [1, 11, 31]. Unhealthy habits have been associated with higher levels of distress and lower levels of well-being [53].
4. The quality of parent–child interaction, as children who experience neglect, abuse, or inconsistent parenting may be at increased risk of mental disorders [46].

On the other hand, some of the protective factors include social support, positive coping skills, physical exercise, and access to mental health resources [25].

Finally, gender and age or developmental period have also been extensively studied as risk factors for emotional disorders. Thus, it can be concluded that the prevalence of anxiety and depression varies according to gender, with problems occurring more frequently in women [7]. For its part, there is a broad consensus in considering that most mental disorders appear around the age of 14. In particular, the precocity of anxiety problems is highlighted, with around 50% of anxiety disorders starting before the age of 18 years and almost 75% before the age of 25 years, while depressive symptoms have a later onset, around 13–20.5 years. Other disorders, such as obsessive–compulsive spectrum disorders or eating disorders, begin around the age of 15 [59].

## Personalised and Tailored Prevention Programs: Strategies for Assigning Participants to Targeted Preventive Interventions

Already in 2007, Crews and colleagues stated that despite the identification of possible risk and protective factors, a crucial objective is the quantification of these factors as well as the determination of which risk factors are specific to which group of disorders (e.g. externalising vs. internalising). Therefore, this has been a matter of interest for more than 15 years since risk and protective factors have been known.

One issue arising from the foregoing is that there is little research on how risk and protective factors should be used to assign to selective or indicated prevention programs and much overlap and arbitrariness in the criteria used. There is, therefore, a need to explore which formula may be the most effective to correctly ascribe to the optimal type of prevention [13]. Thus, in the meta-analysis by Stockings et al. [61], regarding selective prevention interventions, the type of risk used ranged from teenage pregnancy to above-average standard deviation on a personality risk scale, having a close relative with major depression, being a child of divorced parents, having high behavioural inhibition, or having been exposed to war-related trauma, among others. Regarding indicated prevention, the most common types of risks employed were exceeding cut-points for well-established individual measures of depression or anxiety or a combination of several of them (as an example, please see [19]).

To our knowledge, one conclusion is that no research has been conducted to examine which is the best strategy for identifying and assigning individuals to one type of preventive transdiagnostic intervention. Thus, the most appropriate strategy for screening and assigning participants to prevention groups would depend on the specific research question and the available evidence. On the one hand, a purely theoretical-rational strategy may be highly arbitrary and not fully capture the complexity of risk factors for the target population. On the other hand, an empirical strategy that relies solely on previous studies may not consider relevant risk factors that have not been previously investigated. Consequently, the most appropriate and effective strategy for allocating participants to different types of prevention would be one that is based on careful consideration of theoretical and empirical evidence, with a focus on identifying the most relevant and predictive risk factors for the target population; that is, a mixed strategy that combines theoretical and empirical data [27].

## The Present Study

The purpose of this study was to find a theoretical and empirical approach to identify and categorise adolescents seeking psychological help into different levels of preventive intervention for emotional disorders. What has come to be known as personalised prevention proposes innovative methods, and/or designs, such as Latent Profile Analysis (LPA), to identify unique subgroups of individuals who could benefit from prevention interventions [13, 35]. In addition, there are several advantages to using LPA as a “diagnostic” model. First, no specific cut scores are used to identify groups. Rather, all test scores are used simultaneously to determine the probability that an individual is in each latent class. Thus, class membership is based on an individual’s overall pattern of scores. This would remove the need for calculating difference scores at the individual level, which have often been used for emotional disorder identification and are less reliable [43]. Second, this approach moves away from dichotomous diagnostic models to more dimensional ones. Rather than a simple Yes/No decision about whether test scores are consistent with diagnostic criteria, LPA can provide a probability statement of how consistent a person’s test scores are with diagnostic criteria. Further, the model can accommodate differential variation for scores across classes, allowing symptom heterogeneity to be factored into screening decisions. Finally, not all scores are treated equally—some may be more informative for diagnosis than others, depending on latent class characteristics and heterogeneity [13, 43].

Overall, the aims of the present study were (a) to examine the different adolescent profiles in terms of their risk, protective factors, and emotional symptoms; (b) to examine whether gender and age predict adolescents' membership in the risk profiles; and (c) to explore whether there are differences in adolescent outcomes in terms of health and well-being outcomes between the profiles. Concerning our first purpose, and based on previous work highlighting the multifactorial nature of the risk of developing emotional disorders and the main classifications of preventive approaches for mental health, we hypothesise finding between three and five profiles. Moreover, at least two of these profiles will be characterised by opposite levels of risk, resulting in two types of profiles: no risk and extreme risk (high risk, low resilience-high symptomatology). Concerning our second aim, we expect that the adolescents’ age and gender will predict their membership in the higher-risk profiles; that is, there will be more females in the higher-risk groups, and the older they are, the more likely they will belong to the higher-risk groups according to previous research. Finally, about our last aim, we expect to find higher levels of general psychopathology and lower levels of psychosocial adjustment in students belonging to the higher-risk profiles.

## Methods

### Sample

The sample consisted of 1425 adolescents aged between 12 and 18 years (*M* = 14.34, *SD* = 1.76). Out of them, 854 self-reported their gender as female (59.9%), 555 as male (38.9%) and 16 as non-binary gender (1.1%). To determine the sample size, we allocated several observations of 6–10 per variable [64]. Further sociodemographic information can be found in [66].

### Measures

#### 10-Item Connor-Davidson Resilience Scale (CD-RISC-10)

The CD-RISC-10 [6], a shortened version of the Connor-Davidson Resilience Scale [9], comprises 10 items (e.g., “I am able to adapt to changes”), rated on a Likert-type scale with response options ranging from 0 (*not at all*) to 4 (*almost always*). The total score is obtained by the sum of these 10 items. For this study, the Spanish version of the CD-RISC-10 was used, which has previously demonstrated favourable psychometric properties and is recognised as a reliable and valid scale for measuring resilience in adolescents (López-Fernández et al., unpublished work). In the current study, the internal consistency of the scale was high, with a Cronbach’s alpha coefficient (α) and a McDonald's Omega (ω) of 0.84.

#### (Cyber)bullying Scale

The (Cyber)bullying scale [17] was used for this study, which comprises a total of 19 items (e.g., “Have you been sexually harassed via cell phone or the Internet?”), that, added together, provide the total scale score. The response format of this scale was Likert-type from 0 to 4 (from *never* to *always*), indicating the frequency with which the participant has been (cyber) victimised during the last year. The scale has shown good psychometric properties. Adequate reliability indices were also obtained in the sample of this study, with α = 0.81 and ω = 0.80 on the Bullying subscale, and α = 0.86 and ω = 0.87 on the Cyberbullying subscale.

#### Ad Hoc Item on Discrimination

The dichotomous question "Have you ever felt discriminated against for any reason (for example, being part of the LGBTIQ + community, being a migrant, refugee, of another ethnicity, because of your religion or language)?” was added ad-hoc to evaluate the risk of social rejection.

The (Cyber)bullying scale, and the additional question on discrimination comprised the final Social Exclusion Risk Factor latent variable.

#### Fear of COVID-19 Scale (FCV-19S)

As the recruitment was conducted during the pandemic, situations were focused on Covid-19 stressors. The FCV-19S [3] consists of seven items (e.g., “When I see news and publications about COVID-19 in the media, I feel anxious and/or nervous”) answered on a Likert scale from 0 to 4 (from *strongly disagree* to *strongly agree*). The sum of the 7 items provides a total score. A Spanish adaptation was employed to assess the Stress-Related Situations Risk Factor variable [47]. The psychometric properties of the instrument were good for both international and Spanish samples [3, 47], and in this study, we obtained an α of 0.85 and an ω of 0.86.

#### AD HOC Questionnaire Designed Specifically for Unhealthy Lifestyle Habits

A short ad-hoc questionnaire consisting of nine dichotomous questions was developed to identify various health and lifestyle problems (e.g., “I wake up several times during the night”). The questionnaire aimed to assess the Unhealthy Lifestyle Habits Risk Factor variable. This risk factor was calculated using dichotomous responses to the following variables: regular consumption of substances (alcohol, tobacco, or cannabis), daily screen time exceeding four hours, sleep difficulties (such as trouble falling asleep, frequent awakenings during the night, or morning fatigue), and body dissatisfaction (concerns over physical appearance or weight, and physical appearance dissatisfaction).

#### Structured Interview for the Assessment of Expressed Emotion: Child Version (E5cv)

The E5-CV [41], a seven-item structured interview with five response options ranging from 1 to 5 (from *never* to *always*) was used to assess the Parental-Child Interaction Risk Factor variable (e.g., “When some conflictive situation arises that creates stress at home or may foment arguments, my father or mother (either one) gets angry with me and goes as far as insulting me”). The sum of the 7 items provides a total score. Each item covers a dimension of expressed emotion: criticism, generalised hostility, hostile rejection, hopelessness, and self-sacrifice. The scale showed good psychometric properties in Spanish-speaking adolescents with anxiety symptomatology [41]. In this study, an α = 0.77 and an ω = 0.79 were obtained, showing adequate psychometric properties.

#### Revised Child Anxiety and Depression Scale (RCADS-30)

The RCADS-30 [8, 55] is a brief version of the original RCADS-47 [54], which aims to assess symptoms of anxiety and depression in children and adolescents (e.g., “I am afraid if I have to speak in front of the class (face-to-face or through audiovisual media”). This scale is composed of 30 items that are answered on a Likert-type scale scored from 0 to 3 (ranging from *never* to *always*) and comprise six subscales to assess the symptomatology of the following prevalent disorders: major depressive disorder (MDD), panic disorder (PD), social phobia (SP), separation anxiety disorder (SAD), generalised anxiety disorder (GAD), and obsessive–compulsive disorder (OCD). Therefore, from the sum of the items it provides symptomatology scores, not disorder scores, which were used for this study. Previous research has shown that the RCADS-30 has excellent psychometric properties and is a valid version for Spanish populations [48, 48, 49, 49]. In this study, the different scales, following the above order of appearance, also presented excellent internal consistency values: α = 0.85, 0.82, 0.83, 0.70, 0.83, and 0.77, respectively,ω = 0.85, 0.82, 0.84, 0.71, 0.83, and 0.77, respectively.

#### Strengths and Difficulties Questionnaire (SDQ)

The SDQ [22] is an assessment scale for measuring the emotional and behavioural problems of children and adolescents. It comprises 25 items that use a Likert-type response format ranging from 0 to 2 (from *not true* to *certainly true*) (e.g., “I get nervous in new situations, I easily lose my self-confidence”). The sum of the items provides the total score of five subscales: Emotional symptoms, Peer relationship problems, Conduct problems, Hyperactivity/inattention, and Prosocial behaviour. The first two subscales comprise the internalising problems, and the third and fourth subscales comprise the externalised problems. However, only the two externalising subscales and the prosociality subscale were of interest to this study, as the emotional part was already covered by the RCADS-30. It has been translated into multiple languages, including Spanish (www.sdqinfo.org), and the self-reported version has shown adequate psychometric properties for Spanish adolescents [44]. For this study, the following internal consistencies were obtained: α = 0.60 and ω = 0.61 for Conduct problems, α and ω = 0.71 for Hyperactivity/inattention, and α = 0.61 and ω = 0.64 for Prosocial behaviour.

#### KIDSCREEN-10

The KIDSCREEN-10 is a short 10-item questionnaire derived from the original 52-item version (e.g., Have you ever felt lonely?), which assesses the subjective health and psychological, mental, and social well-being of children and adolescents aged 8–18 years, providing a total score of perceived quality of life from the sum of the 10 items [51]. It is answered on a 5-point Likert-type scale, from 1 (*never*) to 5 (*always*). It has versions for different countries, including Spain (https://www.kidscreen.org/english/questionnaires/), which have been shown to have adequate internal consistency indices [51]. Good psychometric properties were also obtained in the study sample, with an α and an ω of 0.85.

### Procedure

The sample was recruited through online advertisements to participate in a survey aimed at young people aged between 12 and 18 years and their parents, to detect and intervene early in those at risk of developing emotional disorders. Those interested participated in the online survey, which was divided into two parts: the first part was to be answered by the adolescent’s parent or guardian, while the second part was to be answered by the adolescents themselves. If, from the answers, we detected that the participants needed help, they could attend a free workshop, where they were enrolled in selective or indicated transdiagnostic preventive intervention workshops (please, see [19, 66, 67]).

The inclusion criteria for recruitment were: (1) living somewhere in Spain and understanding and speaking the Spanish language; (2) having the informed consent of the adolescent and their guardian or legal custodian; (3) being interested in participating in the study.

### Data Analysis

First, a description of the sample and its scores on the different scales administered was obtained. For the continuous scales, the mean score, standard deviation, skewness, and kurtosis were calculated. For the dichotomous scales, the percentage of people who met the "yes" condition (presence of the variable) was calculated. The internal consistency of the scales; that is, their reliability, was also calculated through Cronbach's alpha (α) and McDonald's omega (ω) values. Secondly, both Pearson’s (for continuous variables) and Spearman’s (for dichotomous variables) bivariate correlations between the different variables were calculated to obtain data on the relationship (positive or negative) and magnitude between them. These data are presented in the description of the instruments and in the Supplementary Information (Table SI1).

Thirdly, a LPA was conducted to explore the distribution of adolescents in terms of their protective factors, risk factors, and emotional symptoms. For this purpose, the following variables were used to obtain the profiles: resilience (as a protective factor), risk factor for social exclusion (by bullying and cyberbyllying, and social rejection), stress-related situations (by fear of COVID-19), unhealthy lifestyle habits (by seven unhealthy habits), and parental-child interaction (by expressed emotion), and symptomatology of major depressive disorder, panic disorder, social phobia, separation anxiety disorder, generalised anxiety disorder, and obsessive–compulsive disorder.

Specifically, the LPA was developed from the factor scores of the 11 variables to decrease the effect of measurement errors and considering that the study sample did not follow a normal distribution [30]. With the calculated factor scores, profiles were obtained. To determine the most optimal number of profiles, models with 1 to 8 profiles were obtained, fit indices were calculated for each profile and the best combination of them was selected considering the following criteria: a significant level (*p* ≤ 0.05) of Likelihood Ratio Test (LRT) values, which inform the fit of each model compared to the fit of the model with k-1 profiles; smaller values of Log-Likelihood (LL), Akaike Information Criteria (AIC) and Sample Size Adjusted Bayesian Information Criteria (SSA-BIC), which are indicative of better model fit compared to higher values; and a value close to 1 for entropy. In addition to these fit indices, it was also taken into account that the smallest subgroup within each model should not have too small a percentage of participants (less than 5%), as this would not represent a profile as such, as well as the elbow graph, which visually represents the possible solutions from the AIC and SSA-BIC indices [34, 37, 39].

Once the most optimal profile model was selected, the probability (i.e., odds ratios) of belonging to one profile or another as a function of gender and age variables was estimated by logistic regression analysis using the three-step method (R3STEP function) of MPLUS. Given that the odds ratios showed large magnitude values by sex, an LPA was obtained for each sex, dividing the study sample into girls and boys, and performing the same steps described in this section to obtain a model of 4-profiles for girls and a model of 4-profiles for boys, to allow comparing the two models.

In addition, differences between the profiles obtained in terms of externalising problems (conduct problems and hyperactivity/inattention), prosociality and adolescents' perceived quality of life were also analysed employing an ANOVA and the BCH method of MPLUS [4]. Data were analyzed using the statisticals programs IBM SPSS (version 23), Jamovi (The Jamovi project, 2021) and MPLUS (version 8.7).

## Results

### Latent Profile Analysis

The LPA used to explore the adolescents’ distribution in terms of their protective factors, risk factors, and emotional symptoms showed that the best model was that of four profiles (see Table 1). Firstly, solutions with six to eight profiles were rejected due to the presence of groups with a very small percentage of participants, which may not represent a singular latent profile [37]. Furthermore, the LRT value of these three models did not reach significance, so they had to be discarded (*p* > 0.05). Secondly, fit indices of the 2-, 3-, 4- and 5-profile models were examined to determine the number of profiles that best fit the data. Considering the combination of lower LL, AIC, and SS-BIC values, and high entropy values, the 4-profile solution appeared to be the most optimal (also considering the presence of a group with a very small percentage of participants in the 5-profile solution). The elbow plot supported this possible solution (Supplementary Information: Figure SI1).

**Table 1:** Fit indices for each model of the latent profile analysis

Profiles	Parameters	LL	AIC	SSA-BIC	LRT p	Entropy	% smallest group
1	22	–	30,549.613	30,595.489	–	–	–
2	34	− 15,252.806	23,868.103	23,939.002	0	0.918	49.55%
3	46	− 11,900.051	21,433.987	21,529.910	0	0.907	27.07%
4	58	− 10,670.993	20,418.881	20,539.827	0.0304	0.892	14.21%
5	70	− 10,151.441	19,851.95	19,997.919	0.0304	0.891	7.54%
6	82	− 9855.975	19,618.588	19,789.581	0.3453	0.867	4.08%
7	94	− 9727.294	19,363.135	19,559.151	0.7067	0.848	5.79%
8	106	− 9587.567	19,141.53	19,362.569	0.1399	0.853	3.68%

*LL* Log-Likelihood, *AIC* Akaike Information Criteria, *SSA-BIC* Sample Size Adjusted Bayesian Information Criteria, *LRT* Likelihood Ratio Test

Theoretical considerations were also considered, as well as the purpose of the study; that is, to be able to categorise adolescents seeking psychological help into different types of prevention programs for emotional disorders (selective and indicative). The four-profile solution best described the students’ distribution regarding their risk, protective factors, and emotional symptoms [20, 28, 56].

**Fig. 1:**
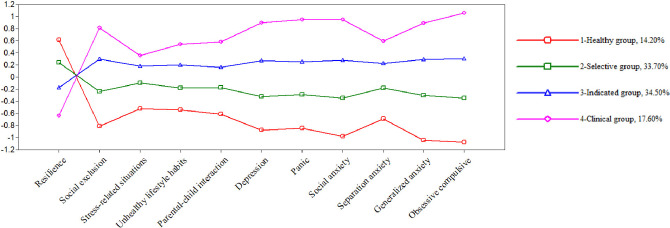
Latent profile analysis: standardised results

As a result of the four-profile model, the following was obtained: 1-A group of adolescents characterised by having the highest scores on the protective factor and the lowest scores on the risk factors and emotional symptomatology, hereafter referred to as the *Healthy group* (14.20% of the sample); 2-A group characterised by having medium–high scores on the protective factor and medium–low scores on the risk factors and emotional symptomatology, hereafter referred to as the *Selective group* (33.70% of the sample); 3-A group characterised by medium–low scores on the protective factor and medium–high scores on the risk factors and symptomatology, hereafter referred to as the *Indicated group* (34.50% of the sample); and 4- A group characterised by the lowest scores on the protective factor and the highest scores on the risk factors and emotional symptomatology, hereafter referred to as the *Clinical group* (17.60% of the sample). This distribution and its descriptive statistics are shown in Fig. 1 and Table 2.

**Table 2:** Means and standard errors (z scores) for the 4-latent profile analysis

	Profiles							
	Healthy group (*n* = 202)		Selective group (*n* = 478)		Indicated group (*n* = 496)		Clinical group (*n* = 249)	
	Mean	SE	Mean	SE	Mean	SE	Mean	SE
Resilience	0.62	0.05	0.24	0.04	− 0.18	0.04	− 0.64	0.04
Social exclusion	− 0.81	0.06	− 0.24	0.05	0.30	0.05	0.81	0.06
Stress-related situations	− 0.53	0.05	− 0.10	0.04	0.18	0.04	0.36	0.05
Unhealthy lifestyle habits	− 0.54	0.04	− 0.18	0.03	0.20	0.04	0.54	0.04
Parental-child interaction	− 0.61	0.05	− 0.18	0.04	0.16	0.04	0.58	0.05
Depression	− 0.88	0.06	− 0.33	0.05	0.27	0.06	0.90	0.05
Panic	− 0.85	0.06	− 0.29	0.05	0.25	0.05	0.95	0.07
Social anxiety	− 0.98	0.07	− 0.35	0.06	0.28	0.06	0.95	0.06
Separation anxiety	− 0.69	0.05	− 0.18	0.04	0.23	0.05	0.59	0.04
Generalised anxiety	− 1.05	0.07	− 0.31	0.06	0.29	0.06	0.89	0.06
Obsessive- compulsive	− 1.08	0.07	− 0.35	0.06	0.30	0.06	1.06	0.07

Odds ratios of the association between the four groups obtained with the LPA and the sociodemographic variables— gender and age—are represented in Table 3. Being female tends to be a criterion for classification into higher psychopathological risk profiles compared to each of the lower risk profiles. Specifically, females were up to 7.16 times more likely to have extreme scores on risk factors and emotional symptomatology and, therefore, to belong to the clinical group. In turn, although with lower odds ratios, being older was also associated with a significantly increased risk of being classified in profiles presenting higher levels of symptomatology and risk factors compared to each of the milder profiles. However, age did not play a role in differentiating the selective and indicated profile, nor the indicated and clinical profile.

**Table 3:** Odds ratio of the association between the psychopathological profiles and sociodemographic variables

Predictors	Profile		OR	95% CI
Gender	1-Healthy	2-Selective	1.69	1.16, 2.46
	1-Healthy	3-Indicated	3.85	2.64, 5.61
	1-Healthy	4-Clinical	7.16	4.43, 11.42
	2-Selective	3-Indicated	2.28	1.69, 3.08
	2-Selective	4-Clinical	4.22	2.81, 6.33
	3-Indicated	4-Clinical	1.85	1.20, 2.84
Age	1-Healthy	2-Selective	1.23	1.11, 1.37
	1-Healthy	3-Indicated	1.33	1.19, 1.47
	1-Healthy	4-Clinical	1.41	1.25, 1.59
	2-Selective	3-Indicated	1.08	0.99, 1.18
	2-Selective	4-Clinical	1.15	1.04, 1.27
	3-Indicated	4-Clinical	1.06	0.96, 1.17

*OR* odds ratio, *95% CI* confidence interval (OR significant when the CI does not contain 1). Gender was coded as 1 = Male/2 = Female; Age ranged from 12 to 18 years clinical profile

**Fig. 2:**
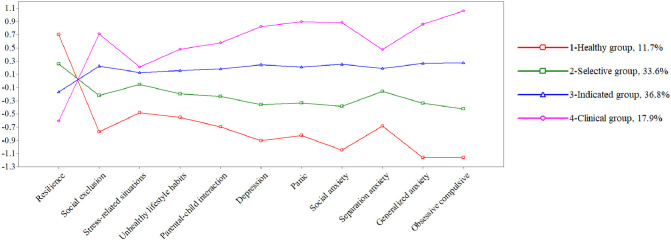
Latent profile analysis results for girls

**Fig. 3:**
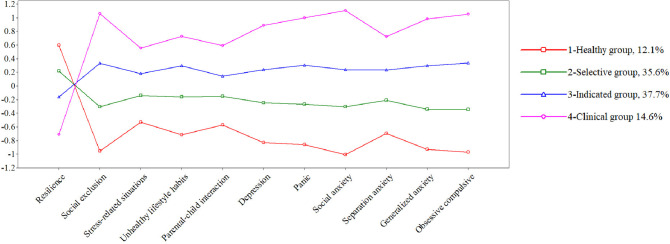
Latent profile analysis results for boys

As the odds ratios for gender were high, LPA was calculated for each gender (Table 4), finding different percentages for males and females. These differences were not large (Figs. 2 and 3), so they are presented only for their usefulness to be considered in future screening programs where the gender perspective is considered when assigning people to different types of prevention interventions.

**Table 4:** Model of 4-LPA profiles for girls and boys

	Parameters	LL	AIC	SSA-BIC	LRT p	Entropy	% smallest group
Girls	58	− 6383.382	12,257.251	12,348.556	0.0348	0.898	11.72%
Boys	58	− 4224.205	8092.260	8158.642	0.0036	0.910	12.07%

*LL* Log-Likelihood, *AIC* Akaike Information Criteria, *SSA-BIC* Sample Size Adjusted Bayesian Information Criteria, *LRT* Likelihood Ratio Test

Finally, the MPLUS BCH method showed statistically significant (*p* ≤ 0.001) differences between the four groups obtained with the LPA and the adolescents' mental health outcomes; that is, behavioural problems, hyperactivity/inattention, prosocial behaviour, and self-perceived quality of life (Table 5). Thus, the four groups differed from each other in all these variables. Specifically, the results revealed that the Clinical group had the highest mean for externalising problem, and the lowest mean for prosocial behaviour and quality of life, just the opposite of the Healthy group. Similarly, the Indicated group showed a higher mean than the Selective group for externalising problems and lower than the Selective group for prosocial behaviour and quality of life.

**Table 5:** Means and standard errors (z scores) for adolescent’s mental health outcomes across latent groups

	Behavioural problems		Hyperactivity/inattention		Prosocial behaviour		Quality of life	
	*M*	*SE*	*M*	*SE*	*M*	*SE*	*M*	*SE*
1-Healthy group (*n* = 202)	− 0.59	0.04	− 0.50	0.03	0.26	0.04	0.87	0.03
2-Selective group (*n* = 478)	− 0.15	0.02	− 0.17	0.02	0.04	0.03	0.29	0.02
3-Indicated group (*n* = 496)	0.19	0.02	0.14	0.02	− 0.09	0.03	− 0.24	0.02
4-Clinical group (*n* = 249)	0.53	0.03	0.41	0.03	− 0.25	0.04	− 0.82	0.03

Equality tests of means across classes using the BCH procedure								
	χ^2^	*p*	χ^2^	*p*	χ^2^	*p*	χ^2^	*p*
1 vs. 3	304.66	< 0.001	272.69	0	48.42	< 0.001	1057.10	0
2 vs. 3	107.54	< 0.001	123.18	0	11.97	0.001	483	0
3 vs. 4	77.11	< 0.001	54.95	0	11.22	0.001	311.65	0
1 vs. 2	92.88	< 0.001	71.27	0	17.99	< 0.001	291.27	0
1 vs. 4	533.99	< 0.001	424.43	0	81.85	< 0.001	1815.66	0
2 vs. 4	344.38	< 0.001	290.51	0	42.89	< 0.001	1298.94	0

*M* Mean, *SE* Standard error, χ^2^ chi-square value higher mean than the Selective group for externalising problems and lower than the Selective group for prosocial behaviour and quality of life

## Discussion

Emotional disorders are common among children and adolescents [50, 58], and and if left untreated can lead to more severe mental and physical health issues [52]. Evidence-based preventive interventions can help reduce emotional disorders and the risk of developing clinical emotional disorders [20, 28, 56]. However, it can be challenging to assign participants to the appropriate intervention program [13, 61], as risk and protective factors for developing emotional disorders are multiple and shared across mental disorders (i.e., [25, 26, 36, 56]). To our knowledge, this is the first study to provide identification of at-risk adolescents, considering not only specific test cut-off scores but also the set of risk and protective factors. This could help to identify and match adolescents at risk of emotional disorders to the appropriate level of preventive interventions using LPA.

Firstly, our results revealed 4 risk profiles, consistent with the multifactorial nature of the risk of developing emotional disorders (e.e., [12, 60]) and the main classifications of preventive approaches for mental health (i.e., [16, 24, 40, 68]). This model allows participants to be classified into four groups based on their protective factors, risk factors, and emotional symptoms: low risk (non-risk participants), moderate risk (suitable for selective intervention), high risk (suitable for indicated intervention), and severe risk group (candidates for clinical referral). These findings align with previous studies examining different psychological variables [13, 43]. Thus, Dishion et al. [13] identified three latent classes of adolescents based on their relationship dynamics with friends and family: healthy, disaffected, and antisocial groups. The latter two groups showed a greater risk for substance use problems, depression, and violence in early adulthood. Findings suggest the potential for tailored family-based interventions to prevent these issues. Niileksela and Templin [43] used Confirmatory Latent Profile Analysis (CLPA) with the Kaufman Test of Educational Achievement, 3rd ed. (KTEA-3) normative sample to identify a latent class consistent with dyslexia across four grade-level groups. This class was also identified in KTEA-3 clinical samples of individuals diagnosed with Specific Learning Disorders of Reading and/or Writing. CLPA may have potential as a diagnostic tool for learning disabilities, but further research is needed.

One implicit and related question concerning the first aim of this paper was to answer whether this theoretically-driven and empirically-derived method would be a better method than traditional methods that employ cut-off points for specific variables (i.e., normative banding scores and cut-off values for SDQ-Emotional problems).

The LPA-based method used here indicates that the participants classified as “clinical” was 17.60%, “high risk-indicated prevention” reached 34.50%, “medium risk-selective prevention” reached 33.70%, and “non-risk/non-clinical was 14.20%. Considering that the sample consisted of “adolescents seeking help for possible risk of developing emotional disorders", the rates are higher than expected, but this is understandable because it is a not normative community-based sample due to the recruitment procedure.

As regards cut-off-based screening studies, we can compare LPA-based percentages to rates of “non-clinical”, “at risk” and “clinical” ranges in this same sample according to the cut-off values for the Emotional problems subscale of SDQ-A in the Spanish population [45]. Following these cut-off scores, we find that 65.8% present “non-clinical” scores (scores below 6), 7.5% “at-risk scores” (equal to 7), and 26.7% are compatible with clinical emotional disorders (above 8). This formula is widely accepted but has pitfalls. For instance, it does not consider risk and protective factors, and emotional symptoms are assessed based on only five items. This may not capture the complexity of emotional disorders. Our findings underscore the need to consider protective/resilience factors when assessing and screening for emotional disorders.

Our LPA-based data are comparable to cut-off points from well-established screening measures such as the SDQ-A in detecting mental health problems. Specifically, our data align with global prevalence rates of mental disorders (13.4%) and emotional disorders (any anxiety disorder = 6.5%, any depressive disorder = 2.6%) reported by Polanczyk et al. [50] and prevalence rates of MDD, dysthymia, and elevated depressive symptoms (8%, 4%, and 34%, respectively) among adolescents aged 10 to 19 reported by Shorey et al. [58]. Our findings are consistent with the criteria used by Goodman in the original version of the SDQ and with empirical findings on the detection and prevalence of mental health problems [2, 23]. These data support the notion that around 10% (scores above the 90th percentile) of children and adolescents in community-based samples exhibit mental health problems, while another 10% (scores between the 80th and 90th percentile) have borderline problems, based on threshold values for clinical, at-risk, and non-clinical categories [45].

Our study's second objective was to explore whether adolescents' age and gender could predict their membership in higher-risk profiles. We hypothesised that females and older adolescents would be more likely to belong to higher-risk groups, and our findings supported this hypothesis. Specifically, we found more females in the higher-risk groups, and the older the adolescent, the more likely they were to belong to a higher-risk group. These results are consistent with previous literature, which has consistently found that the female gender and the adolescent developmental period increase the likelihood of belonging to groups at risk for developing emotional disorders [7, 59]. Therefore, our study supports the idea that prevention programs for emotional disorders should be designed with a gender and age perspective in mind. By tailoring interventions to the specific needs and risk factors of different gender and age groups, we may effectively reduce the risk of developing emotional disorders in adolescents.

Finally, concerning our last objective, we aimed to investigate the relationship between higher-risk profiles and levels of general psychopathology and psychosocial adjustment. We hypothesised that students in the higher-risk profiles would exhibit more psychopathology and lower levels of psychosocial adjustment. Our findings supported this hypothesis and revealed that the Clinical and Indicated groups had, as expected, higher levels of externalising problems and lower levels of prosocial behaviour and quality of life compared to the Healthy and Selective groups according to the main classifications of prevention interventions [16, 24, 40, 68]. These results also align with previous studies [52] that suggest that undetected emotional disorders tend to persist and become chronic, leading to the development of other disorders, which can ultimately result in adverse physical health outcomes and increased mortality risk. The implications of these findings underscore the importance of identifying and treating emotional disorders in adolescence to improve quality and length of life and reduce healthcare costs associated with physical illness in line with Garcia-Lopez [18]. Therefore, the design of prevention and intervention programs should consider the severity and implications of higher-risk profiles to improve psychosocial functioning and mental health outcomes in adolescents. These data also are consistent with Dishion et al. [13], who, using LPA to categorise adolescents based on their observed relationship dynamics with friends and family, found that disaffected and antisocial groups showed a greater risk for substance use problems, depression, and violent offending in early adulthood compared to the healthy relationship group.

Some limitations should be noted. First, one limitation is that we did not use parental information. However, adolescents are typically considered the best informants for detecting emotional problems. This was because we only counted those who had completed all the measures considered, and those who only had the parent report (SDQ-P) were eliminated. Secondly, it is worth asking why we used the variables employed in the LPA and not others. In this regard, we note that we used the measures that theoretically made the most sense according to the systematic reviews analysed and the theoretical models reviewed, as well as following the principle of not including in the equation those variables or measures with high correlations between them, indicating multicollinearity; in other words, variables that overlap or measure the same or very similar constructs. This is the case of RCADS and SDQ-A Emotional, for example. In addition, many other combinations were tested, and the most theoretically appropriate and the one that showed the most consistent results with the accumulated evidence was chosen.

### Conclusions

In conclusion, this study highlights the potential of personalised prevention approaches to identify and categorise adolescent participants into different levels of preventive interventions for emotional disorders based on their risk and protective factors. Such an approach may help prevent emotional disorders from becoming chronic and associated with other disorders, leading to more severe mental and physical health issues.

The theoretically-driven and empirically-derived formula of this study allows us to improve the system of screening, detection, selection, and assignment of participants at risk of developing emotional disorders to different modalities of preventive interventions. The findings provide important insights for developing and implementing targeted prevention programs for adolescents with emotional disorders. This approach could facilitate the identification of at-risk adolescents and help allocate them to personalised and tailored prevention programs that match their specific needs. This could be this work’s main applied contribution that we can transfer to society.

## Summary

This study aimed to address the challenge of assigning adolescents to appropriate intervention programs for emotional disorders by identifying and categorizing them into different severity levels. Utilizing data from 1425 Spanish adolescents a Latent Profile Analysis (LPA) was conducted to discern subgroups based on emotional symptom severity, risk, and resilience factors. The analysis revealed four distinct profiles: those at low risk (considered emotionally healthy), moderate risk (appropriate for selective interventions), high risk (indicating a need for indicated interventions), and severe risk (suggesting clinical referral). Notably, older age and female gender were predictive of higher risk clusters. Additionally, variations in psychopathology and health-related quality of life were observed across these clusters. The findings underscore the potential of LPA in identifying at-risk adolescents for emotional disorders, offering insights for personalized and tailored prevention programs tailored to meet individual needs.

## Supplementary Information

Below is the link to the electronic supplementary material.

Supplementary file1 (PDF 193 kb)

## Data Availability

The data, materials and analysis code for this study are not available for legal and ethical reasons: they were not expressly requested in the informed consent of the participants or in the ethics committee. Under justified reasons, they could be requested from the corresponding author.
