# Supplementary material for: Identifying Adolescents at Risk for Emotional Disorders with Latent Profile Analysis: A Personalized, Transdiagnostic Preventive Intervention
Source: Child Psychiatry Hum Dev. 2024 Apr 12;57(1):124–37. doi: 10.1007/s10578-024-01689-z (PMC12971773; doi:10.1007/s10578-024-01689-z)

Identifying adolescents at risk for emotional disorders with latent profile analysis: A personalized, transdiagnostic preventive intervention

Child Psychiatry & Human Development

José A. Piqueras, Raquel Falcó, Pilar Rico-Bordera*, Josefa Canals, Lourdes Espinosa-Fernández, Manuel Vivas-Fernández, & Luis-Joaquín Garcia-Lopez on behalf of PROCARE team

Table SI1

Descriptive statistics, reliability indices, and correlations among adolescent variables

	1	2	3	4	5	6	7	8	9	10	11	12	13	14	15	16	17	18	19	20	21	22	23	24	25
1	1																								
2	-.27**	1																							
3	-.25**	.35**	1																						
4	-.26**	.28**	.33**	1																					
5	-.12**	.16**	.10**	.08**	1																				
6	-.12**	.17**	.17**	.09**	.03	1																			
7	-.07**	.13**	.13**	.07**	.01	.43**	1																		
8	-.14**	.12**	.15**	.08**	.02	.38**	.52**	1																	
9	-.17**	.16**	.15**	.11**	.00	.09**	.06*	.05*	1																
10	-.12**	.08**	.06*	.08**	.03	.05	.05*	.04	.07**	1															
11	-.26**	.17**	.16**	.10**	.09**	.11**	.10**	.09**	.09**	.19**	1														
12	-.25**	.14**	.15**	.17**	.09**	.08**	.08**	.09**	.13**	.17**	.33**	1													
13	-.19**	.19**	.13**	.17**	.12**	.14**	.13**	.13**	.11**	.20**	.23**	.17**	1												
14	-.34**	.23**	.26**	.23**	.16**	.11**	.09**	.10**	.19**	.13**	.26**	.30**	.25**	1											
15	-.42**	.23**	.25**	.27**	.11**	.14**	.12**	.14**	.18**	.10**	.26**	.31**	.21**	.63**	1										
16	-.57**	.21**	.33**	.30**	.18**	.11**	.10**	.13**	.20**	.16**	.29**	.35**	.24**	.45**	.47**	1									
17	-.43**	.38**	.20**	.25**	.28**	.14**	.11**	.14**	.15**	.12**	.28**	.26**	.19**	.30**	.32**	.52**	1								

	1	2	3	4	5	6	7	8	9	10	11	12	13	14	15	16	17	18	19	20	21	22	23	24	25
18	-.47**	.25**	.20**	.27**	.31**	.06*	.04	.06*	.13**	.12**	.23**	.27**	.19**	.38**	.38**	.59**	.49**	1							
19	-.30**	.03	.28**	.17**	.29**	.01	-.04	.01	.02	.08**	.17**	.18**	.13**	.24**	.20**	.47**	.25**	.41**	1						
20	-.42**	.33**	.17**	.21**	.37**	.11**	.08**	.09**	.17**	.14**	.22**	.25**	.22**	.35**	.33**	.41**	.40**	.46**	.37**	1					
21	-.47**	.37**	.23**	.25**	.26**	.11**	.12**	.14**	.18**	.18**	.27**	.28**	.24**	.33**	.33**	.50**	.55**	.52**	.37**	.66**	1				
22	-.33**	.28**	.25**	.20**	-.01	.17**	.16**	.14**	.15**	.12**	.11**	.14**	.26**	.20**	.23**	.30**	.23**	.19**	.17**	.26**	.32**	1			
23	-.35**	.23**	.19**	.17**	.05	.13**	.13**	.17**	.16**	.12**	.14**	.16**	.22**	.20**	.23**	.32**	.27**	.23**	.20**	.29**	.38**	.53**	1		
24	.25**	-.05*	-.07**	-.06*	.14**	-.04	-.04	-.02	-.14**	-.04	-.04	-.07**	-.14**	-.07**	-.10**	-.16**	-.05	-.03	-.02	-.08**	-.10**	-.33**	-.21**	1	
25	.64**	-.31**	-.28**	-.31**	-.17**	-.18**	-.16**	-.19**	-.19**	-.19**	-.32**	-.39**	-.34**	-.48**	-.54**	-.66**	-.51**	-.48**	-.29**	-.48**	-.53**	-.34	-.37**	.25**	1
M	2.38	0.29	0.12	19.40%	0.97	5.60%	4.40%	5.90%	45.20%	26%	25.50%	43.20%	1.81	48.80%	34.04%	1.12	0.54	1.49	0.59	1.39	0.83	0.45	0.96	1.59	3.34
SD	7.71	0.48	0.23	—	0.81	—	—	—	—	—	—	—	0.65	—	—	0.77	0.61	0.83	0.59	0.79	0.71	0.38	0.50	0.36	0.77

Note. 1 = Resilience; 2 = Bullying; 3 = Cyberbullying; 4 = Social rejection; 5 = Fear of COVID-19; 6 = Alcohol use-related problem; 7 = Cannabis use-related problem; 8 = Tobacco use-related problem; 9 = Internet use-related problem; 10 = Difficulties falling asleep; 11 = Problems waking up at night; 12 = Unrefreshing sleep; 13 = Family Expressed Emotion; 14 = Concerns over physical appearance/weight; 15 = Physical appearance dissatisfaction; 16 = Depression; 17 = Panic; 18 = Social anxiety; 19 = Separation anxiety; 20 = Generalised anxiety; 21 = Obsessive-compulsive; 22 = Conduct problems; 23 = Hyperactivity/inattention; 24 = Prosocial behaviour; 25 = Quality of life; * $p < .05$. ** $p < .01$.

Figure SI1

Elbow plots for latent profile analysis

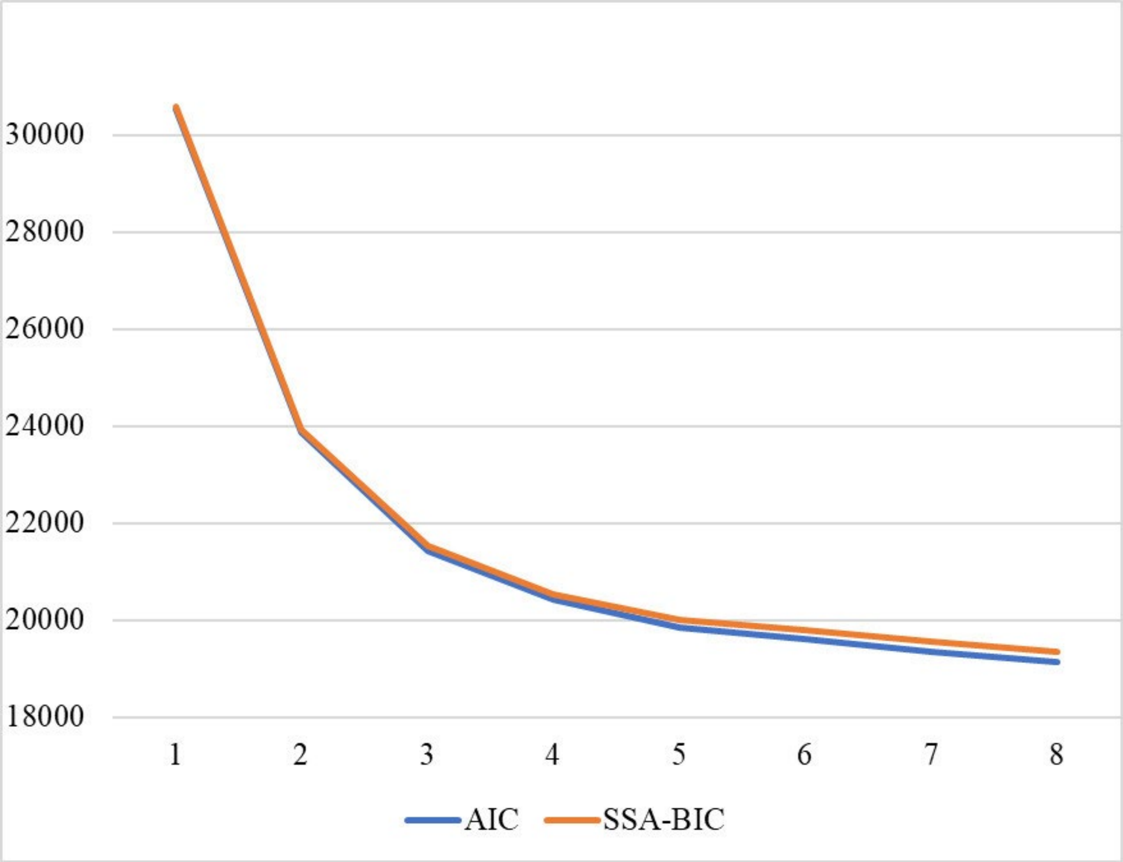

Supplement: Supplementary file 1 — Supplementary file1 (PDF 193 kb) [file 10578_2024_1689_MOESM1_ESM.pdf]
